# Traumatic pulmonary pseudocyst: a rare complication of blunt thoracic injury

**DOI:** 10.11604/pamj.2019.32.180.18592

**Published:** 2019-04-11

**Authors:** Ouissal Aissaoui, Rachid Alharrar

**Affiliations:** 1 Surgical ICU and Resuscitation Department P33, University Hospital of Ibn Rochd, Casablanca, Morocco

**Keywords:** Traumatic pulmonary pseudocyst, traumatic pneumatocele, blunt chest trauma

## Image in medicine

Traumatic pulmonary pseudocyst is a rare and underreported complication of blunt chest trauma. It represents air trapped within a pulmonary laceration. It generally appears on radiographs as a thin-walled, air-filled cavity, with or without air-fluid levels. It is very important for emergency physicians and pediatricians to be familiar with this condition because it generally represents a benign, self-limited condition that only requires observation. We report a case of a young man admitted in our ICU for thoracic injury consecutive to a motor vehicle accident. The chest X-Ray showed multiple large thin-walled cystic lesions. The Chest CT identified several large cavities on the right lung confirming the diagnosis of traumatic pulmonary pseudocysts. He was treated by oxygen therapy and analgesia. His condition was relatively stable and improved after few days. He was discharged 10 days later and a follow-up chest CT showed spontaneous resolution of the pseudocyst.

**Figure 1:**
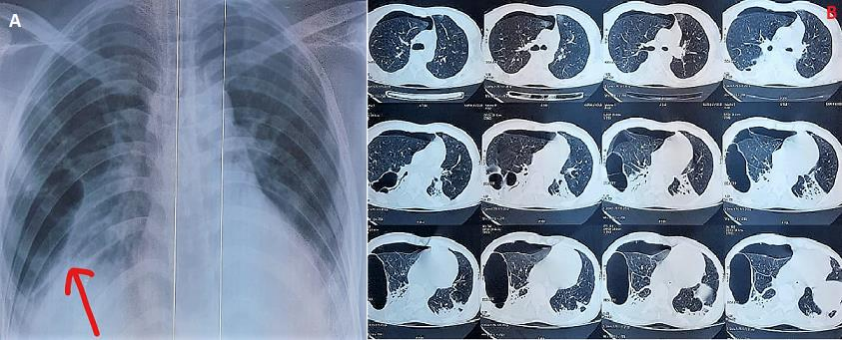
A) chest X-Ray: thin walled cavities; B) chest CT scan: multiple and large air filles cavities in the right lung

